# Women’s experiences of treatment for mastitis: A qualitative study

**DOI:** 10.18332/ejm/137356

**Published:** 2021-06-29

**Authors:** Isabell K. Tøkje, Solfrid L. Kirkeli, Lena Løbø, Bente Dahl

**Affiliations:** 1 Women’s Clinic, Haukeland University Hospital, Bergen Hospital Trust, Bergen, Norway; 2 Centre for Women’s, Family and Child Health, Faculty of Health and Social Sciences, University of South-Eastern Norway, Borre, Norway

**Keywords:** breastfeeding, qualitative, inflammation of the breast, non-medication treatment, mastitis

## Abstract

**INTRODUCTION:**

Mastitis (inflammation of the breast) occurs in 10–20% of breastfeeding women. Different levels of the health service meet the needs of women in different ways, and treatment procedures vary at a local level. As maternity care evolves, with a greater need for efficiency, and the treatment for women is also changing. The purpose of the study is to investigate women’s experiences of treatment for mastitis in the interface with the health service.

**METHODS:**

The study has a qualitative design. It was conducted in 2018 and included semi-structured interviews with 11 women living throughout Norway who received treatment for mastitis. The data material was analyzed using systematic text condensation.

**RESULTS:**

The analysis resulted in three themes. The first theme was the interaction with healthcare personnel and related to being treated in a caring manner. The second concerned the women’s experience of feeling overwhelmed by pain and having to depend on help. The third theme related to the hospital stay and the women’s experiences of how random factors govern the treatment of the disease.

**CONCLUSIONS:**

The study shows that women who were treated for mastitis were at risk of being admitted to hospital wards characterized by a lack of knowledge about mastitis, insufficient or flawed treatment competence, and an absence of necessary equipment. Treatment should be organized in a way that enables women to be placed in wards where staff have knowledge and experience, such as maternity and gynaecological wards, as well as associated outpatient clinics.

## INTRODUCTION

Breastfeeding has proven health benefits for mother and child^1-4^, and despite a slight decline in breastfeeding rates, most Norwegian women breastfeed their infants^3^. For most women, breastfeeding does not entail any major complications, but approximately 10–20% of women develop inflammation of the breast (mastitis)^5-7^.

Mastitis is an inflammatory reaction, either with or without a bacterial infection. The symptoms are red, painful, hot, swollen breasts, and sometimes fever, chills and flu-like symptoms. Inflammatory (non-bacterial) mastitis is caused by interstitial oedema trigged by milk stasis^5^. Bacterial mastitis is caused by bacteria spreading to the milk ducts through cracks or fissures in the skin surrounding the nipples, often in the form of yellow staphylococci. Both types of mastitis require non-medication treatment and analgesics, but bacterial mastitis may also require treatment with antibiotics^5-7^. For the first 24 hours of treatment for mastitis, the woman will be in contact with the primary healthcare service, after which she will normally be referred to the specialist health service^7,8^. Mothers experiencing malaise, a decline in general health and breast pain may require relief and help with their infant as well as with milk drainage during the acute phase of the illness. Some women choose to stop breastfeeding because the symptoms become so severe^9^.

Studies describe various pain-relieving and therapeutic interventions in the non-medication treatment of mastitis^9-11^. Frequent breastfeeding, a good latch, hand expressing, pumping and massage are especially important until the correct diagnosis is made, normally during the first 24 hours. However, it is essential to fully drain the breast throughout the entire course of treatment for mastitis, both for bacterial and inflammatory mastitis. This involves emptying the breast every two hours during the day and at least twice during the night^5^. Studies also show that women with mastitis experience acute malaise accompanied by breast pain, negative thoughts and ambivalence towards whether they will be able to continue breastfeeding^12,13^. This can further complicate the situation, as some women associate successful breastfeeding with being a good mother^12^. For women who have experienced trauma or stress related to childbirth, successful breastfeeding can be crucial to counteracting the negative emotions stemming from this experience^14^.

The purpose of the study was to investigate women’s experiences of treatment for mastitis in the health service.

## METHODS

### Design and sample

A qualitative design was chosen, since the purpose of the study was to explore the women’s perceptions and experiences of treatment for mastitis^15,16^.

We recruited a convenience sample^17^ using a social networking site. ‘Ammehjelpen’ (a breastfeeding assistance service) shared the post, and 33 women contacted the first author via email. Three primiparous women and eight multiparous mothers who received inpatient or outpatient treatment for mastitis within the first year of childbirth were included. All the women breastfed their infant during the period of illness. We focused on women’s experiences with non-medication and pain-relieving interventions for treating mastitis, but also included women who had received antibiotics without focusing on the effect of this. Twenty women were treated solely by a general practitioner and did not therefore meet the inclusion criteria. Two women were excluded because they were selected for surgery at the first consultation. This resulted in a sample consisting of 11 women aged 26–43 years who had received treatment for mastitis in the period December 2016 to December 2017.

Semi-structured individual interviews were used to obtain data. Three of the authors (IKT, SLK, LL) conducted interviews individually in the period February to May 2018. Nine participants were interviewed by telephone and two were interviewed in-person in a meeting room at a public library. The interviews lasted between 11 and 45 minutes (average 35 min), and audio recordings were made, which were transcribed after each interview. The interviews produced rich data that could shed light on the subject of the study, and we chose to stop recruiting participants after we identified a pattern of repetition in the themes that the women described.

An experiential interview guide was used, consisting of four open-ended questions relating to the purpose of the study^15^. The questions concerned the women’s experiences of having suffered from mastitis, the help they received when they were diagnosed with mastitis and their reflections related to the help they received. The participants spoke freely about the main issues but were asked questions if there was a need for clarification or elaboration.

### Data analysis

Data were analyzed collectively using systematic text condensation (STC), a four-step method for thematic crosscase analysis^17^. In the first step (from raw data to themes), the interview transcripts were read by all the authors in order to form an overall perspective. They were then reviewed by two authors jointly to identify preliminary themes. These were discussed by all authors as a group, and agreement was reached. In the next step (from themes to code groups), the interviews were read again and units of meaning, i.e. fragments of text containing information about the research question, were identified in the text and sorted into code groups. According to Malterud^17^, the coding process implies marking the meaning units with a code or label, noting their commonalities and differences and sorting them into groups with related meaning. In the third step (from code to meaning), we considered one code group at a time and organized the content into different subgroups describing different aspects of the phenomenon. In this step of the analysis process, the subgroups became the focus of our attention. We reduced the content in each subgroup into a condensate: ‘an artificial quotation maintaining, as far as possible, the original terminology applied by the participants’^17^. Finally, in step four (from condensation to descriptions and concepts), we synthesized the condensates into an analytical text. Quotes illustrating the text were included. An example from the analysis process is given in Table 1 and an overview of code groups and subgroups is presented in Table 2.

**Table 1:** Example of the analysis process (eleven Norwegian women’s experiences of being treated for mastitis in 2018)

*Step 1* *Units of meaning*	*Step 2* *Code group*	*Step 3* *Subgroups*	*Step 4* *Theme/analytical text*
‘The worst thing was that one time it took eight hours from the time my breasts were emptied to the next time they were emptied because they didn't have a pump available. They had to go looking for a pump in another department. It felt like it took forever. I lay there and just got worse and worse, and I cried and cried and was in so much pain. Eight hours!’ ‘I fought, howled and screamed and said I did not want to return to the infection control ward. I said it didn't go well the last time, no one had any idea what to do, it took ages between each time my breasts were emptied and I got worse and worse instead of getting better and better.’ ‘How often should I express by hand? They all told me something different. Some thought I should do it every two hours and others thought every three hours, and some thought every four hours. My head was swimming from all the conflicting information.’	Random treatment	No equipment or unavailable equipment Randomly selected ward Lack of competence/knowledge	The hospital stay: random factors govern the treatment

**Table 2:** Overview of code groups and subgroups (eleven Norwegian women’s experiences of being treated for mastitis in 2018)

*Code groups*	*Subgroups*
Interaction with healthcare personnel: being treated in a caring manner.	Belittled during first contact. Good follow-up was crucial to women’s experiences. Passed like a parcel between different units.
Experience of illness: feeling overwhelmed by pain and depending on help.	Varying experiences of pain relief. Mental strain in women with active cases of mastitis. Feelings of guilt in relation to attachment to infant.
The hospital stay: random factors govern the treatment.	Lack of competence/knowledge among staff. No equipment or unavailable equipment. Randomly selected wards.

### Ethical considerations

The study was conducted in accordance with the Declaration of Helsinki^18^ and considered by the Regional Committees for Medical and Health Research Ethics (REC) to fall outside the scope of the Health Research Act (REK: 2017/2519 A). The study was approved by the Norwegian Centre for Research Data (NSD: 57945).

Prior to the interviews, the participants were sent information letters and consent forms. They were informed that they could withdraw from the study without any consequences for them before completion of the data processing. Participants agreed orally by telephone or in writing by email.

## RESULTS

The analysis resulted in three themes. The first was the interaction with healthcare personnel and related to being treated in a caring manner. The second concerned the women’s experience of feeling overwhelmed by pain and their dependence on help. The final theme related to the hospital stay and the women’s experiences of how random factors govern the treatment.

### Interaction with healthcare personnel: being treated in a caring manner

Many women felt they were not believed or taken seriously when they contacted a general practitioner, the out-of-hours medical service or outpatient clinic for symptoms of mastitis; and some found that they had to convince healthcare personnel of their condition:

*‘I felt that the people I spoke to on the phone the first time belittled me. They didn't think it was mastitis, but I was sure, because I had had it before! They decided to believe me in the end, and then they gave me the information I needed. And it was a bit like, yeah, that's what I said.’* (Participant 3)

In contrast, women who were referred to hospital for further assessment found that they were well taken care of and taken seriously. Several emphasized that personal follow-up helped make the hospital stay a positive experience. One woman described how the interaction with an empathetic doctor and a midwife who cared for her continuously throughout her hospital stay saved her from feeling like a parcel being passed around in the system. However, it was not important who carried out the treatment:

*‘There was a wonderful male nurse whose wife had had mastitis. He had helped her to express her milk by hand, so he knew how to do that. He came and helped me, and then he taught the others at work how to do it.’* (Participant 6)

However, several participants found that healthcare personnel were busy and did not understand the condition. Some did not dare to ask for help for fear of being a burden, while others felt they were brushed off when they asked for help, and wondered if they could take more responsibility themselves.

*‘But then I also think that I could've been better at asking for help, but it's not always easy to ask for help when you need it.’* ( Participant 10)

### Experience of illness: feeling overwhelmed by pain and depending on help

The women said that having mastitis was a terrible experience. The physical pain was not localized only to the breasts, it ran through the entire body. They described how a high temperature and lack of energy led to a general feeling of being unwell, and how they were unprepared for the pain. They wondered why no one had told them that mastitis was so painful and thought there was something wrong with their own pain threshold. The pain also led to tenderness when touched and made breastfeeding difficult:

*‘It was much worse than giving birth. It was painful, uncomfortable, it was my whole body … it was like my whole body was… I was dizzy. I had no strength. I had a high temperature, it was terribly, terribly painful.’* (Participant 5)

The women said that pain relief was important, especially heat treatment, which reduced pain when touching and draining the breasts. One of the women said that she felt in her whole body how important it was to express the milk by hand and drain her breasts. Some said it helped to sit in the shower and warm up their breasts before a massage, while others found that warm wraps in the form of rice bags or cotton wadding were effective. They found that heat treatment given in combination with analgesics made it easier to breastfeed their infant and reduced fever and general malaise. Help in correcting the latch or trying out different breastfeeding positions in tandem with the use of analgesics also reduced the pain considerably. However, all interventions required proper guidance and practical help, which could be time consuming:

*‘The fact that the midwives sat down and helped me express the milk by hand and that I wasn't left there on my own. But personal follow-up, someone sitting down with me and showing me how to squeeze out the milk by hand, that was the best thing really.’* (Participant 7)

Several participants experienced a mental strain when breast pain made it difficult to hold their infant close to them. They felt guilt about their attachment to the child, especially in cases where it was not long after they had given birth. Being locked in ‘pain hell’ was detrimental to their attachment to the infant, but the women were nevertheless grateful to have their child with them. One woman said it hurt just to move her arms because of the size of her breasts. Taking care of the child was unthinkable:

*‘Your head felt completely frazzled, new baby, new life situation and a baby to take care of. Then I couldn't take care of him and it was just sad; a sad feeling where you felt that you couldn't take care of your own baby because you couldn't even take care of yourself.’* ( Participant 8)

The women found that healthcare personnel lacked knowledge about the use of medications. Some had searched the internet on the topic and found information about which medications were compatible with breastfeeding. However, the healthcare personnel had a restrictive approach to the use of medication due to the risk of it transferring to the breast milk, and some were unsure which medications were compatible with breastfeeding:

*‘She said that it would be okay for me to take some paracetamol and see if it took away the fever and some of the pain. But warm wraps and a hot shower were sometimes the best relief for pain.’* (Participant 9)

### The hospital stay: random factors govern the treatment

At smaller hospitals, the women often experienced being admitted to wards with expertise in women’s health, and felt they received support and guidance from qualified personnel with expertise in the treatment of mastitis. Women who received treatment in maternity wards at larger hospitals described similar experiences, while women who were admitted to infection control wards or wards with spare capacity at these hospitals said that they encountered healthcare personnel with no knowledge, experience or skills in the treatment of mastitis. One woman describes the information she received about the hand expression of milk:

*‘How often should I express by hand? They all told me something different. Some thought I should do it every two hours and others thought every three hours, and some thought every four hours. My head was swimming from all the conflicting information.’* ( Participant 4)

Wards also lacked the necessary equipment. A woman was wheeled into an infection control ward in a wheelchair and left to fend for herself despite barely being able to hold her head up. She said her husband had to ask staff to find a bed and a pump. Another woman described how she was left in bed with overly full breasts because it took so long to borrow equipment from other wards:

*‘One time it took eight hours from the time my breasts were emptied to the next time they were emptied because they didn't have a pump available. They had to go looking for a pump in another department. It took forever. I lay there and just got worse and worse, and I cried and cried and was in so much pain. Eight hours ….’* (Participant 1)

The women expressed frustration at being moved between different wards, and that the staff they met had neither expertise in the treatment of mastitis nor breastfeeding. Many found that healthcare personnel followed the ward’s treatment procedures without their needs being taken into account, while one woman experienced the opposite:

*‘The midwife saw the problem and she acted immediately. She gave me so much advice, so much guidance. She came in her spare time and helped me. She saw how much pain I was in, she saw that I was not being treated as I should have been. She came to see me when she finished her shift. She sat next to me and emptied my breasts. It took a long time but she managed it. The only one who managed it.’* ( Participant 6)

## DISCUSSION

For breastfeeding women, mastitis is a very painful diagnosis that requires effective pain relief, thoughtful care and good advice. The women in the study also emphasized the importance of continuity, individually tailored treatment and consistent information, but were sometimes faced with staff who lacked knowledge and interest in their situation. When hospital wards also lacked the necessary equipment, some felt that random factors governed the treatment.

Pain was a recurring theme in the data material. In order to assess pain, it is important to listen to the women’s subjective experiences^19^. When pain-relieving interventions were not initiated, the women considered this to be due to the healthcare personnel’s lack of competence. Women who were treated by knowledgeable healthcare personnel found that treatment interventions were initiated, such as heat packs, massage and the hand expression of breast milk. Draining an inflamed breast will relieve symptoms by reducing swelling and pressure, which in turn will relieve pain^11^. However, several women found that they did not receive pain-relieving interventions or analgesics, and felt that random factors determined the treatment. Research^11,20^ shows that breastfeeding problems alone or in tandem with other physical health problems, such as pain from mastitis, can be associated with a low mood in the postnatal period. For some women in the study, the pain became so great that they were unable to take care of their infant, which became a mental strain. The women found this difficult, and some felt guilt and grief. It is therefore important that women with mastitis receive good pain relief that enables them to take part in the care of their child^21^. Our results also imply that women who receive treatment for mastitis find that healthcare personnel are unsure which medications are compatible with breastfeeding, and this leads to conflicting advice. Information can be interpreted in different ways, which makes it difficult to establish what is correct^22^. To ensure optimal treatment, it is therefore important that healthcare personnel avoid misinforming women or giving conflicting advice^23,24^.

It was crucial to the women that healthcare personnel were available and competent, and focused on their needs. Similar results are described by Wheelan and Kearney^22^. Focusing on individual needs can strengthen the experience of co-determination, and women who are seen in person achieve better results and have a better experience with the treatment than women who only have telephone contact^25,26^. Involving women in the choice of treatment methods can help them feel reassured and reduce stress levels, and is appropriate as long as the methods are not indefensible or at odds with applicable legislation or professional guidelines^27^. Furthermore, being taken seriously can impact on the patient’s own efforts and the outcome of treatment^28^. A busy ward can represent a barrier to women receiving the support and help they need^22,29^. Spending time with a patient can help reassure them, as the competence of the staff becomes more visible and improves the woman’s experience^28,30^. Our findings support this.

Some of the women found that if they did not fit into the ward’s routines, they were considered a burden and largely left to themselves. Health care must be organized so that healthcare personnel can carry out their work in accordance with their statutory duties, but studies show that healthcare personnel who have to comply with many guidelines and regulations can find it difficult to adapt the treatment to the women’s needs^22,25^. The Norwegian Society for Gynecology and Obstetrics^5^ and Oslo University Hospital’s advisory unit for breastfeeding^6^ have published guidelines for the treatment of mastitis, but procedures for the correct treatment of mastitis should be available in all hospitals.

The knowledge that emerged in the study is relevant to all healthcare personnel working with breastfeeding women. Increased understanding of women’s experiences of treatment for mastitis can facilitate the development of a well-adapted treatment provision. This is important for women’s ability to continue breastfeeding and for preventing the condition from becoming a mental strain. Tailored treatment requires good procedures to be in place in the treatment units, healthcare personnel to have knowledge of the condition, and the necessary equipment for treating the condition to be available.

### Strengths and limitations

The choice of qualitative design with individual interviews and thematic analysis was appropriate, as a literature search did not uncover previous studies that shed light on women’s experiences with the topic. Nine of the eleven interviews were conducted by telephone at the request of the participants. Although this affected our ability to observe non-verbal communication in the interview situation, the interviews produced rich data that helped to elucidate the subject of the study. One of the interviews lasted only 11 minutes, but nevertheless provided relevant information and was therefore included in the study. Three of the authors conducted the interviews, but used an interview guide to ensure that all women were asked the same questions.

The women lived in different parts of Norway and had received different treatment for mastitis. Eight of the women were multiparous mothers with previous breastfeeding experience, and some also had previous experience with mastitis. Although this strengthens the study, we are aware that experience is a subjective phenomenon and that some of the experiences described happened a year before the interview. The use of social media has probably also led to a somewhat uniform sample, in that only women who were interested in sharing their experiences were included. This may affect the validity of the study^17^.

The authors of the article are or have been employed in the specialist health service and have experience in treating this patient group, including pain relief, non-medication treatment and help to ensure satisfactory nutrition to the child. When we decided to explore the topic, we discussed the challenges that our own knowledge of and experiences with mastitis treatment could entail in the design of the interview guide and in the interview situation. We also discussed whether we could risk including participants that we had treated ourselves, and agreed not to interview these ourselves. However, this issue proved to be irrelevant, as participants volunteered from all over Norway.

## CONCLUSIONS

The study shows that some women who have been admitted to the specialist health service for the treatment of mastitis found the non-medication interventions to be unsatisfactory, while others felt they were well taken care of. The negative experiences can be linked to the experience of being admitted to wards characterized by a lack of knowledge about mastitis, insufficient or flawed treatment competence, and an absence of necessary equipment. The treatment of this patient group should be organized in a way that enables women to be placed in wards where the staff have knowledge and experience in treating mastitis, such as maternity and gynaecological wards, as well as associated outpatient clinics.

## Data Availability

The data supporting this research cannot be made available as participants were informed that data would be available for the researchers only.

## References

[1] Nilsson I, Busck-Rasmussen M (2021). Amming: en håndbog for sundhedspersonale.

[2] (2021). Breastfeeding. Health Topics.

[3] (2016). Spedbarnsernæring: Nasjonal faglig retningslinje.

[4] Victora CG, Bahl R, Barros AJ (2016). Breastfeeding in the 21st century: epidemiology, mechanisms, and lifelong effect. Lancet.

[5] Fossum B, Brabrand K, Myr R, Prestaasen R (2020). Amming/morsmelk/mastitt og abscess. Veileder i fødselshjelp.

[6] Brystspreng, mastitt og abscess (2019). Medisinske brystkomplikasjoner ved amming. Nasjonal kompetansetjeneste for amming (NKA).

[7] Nordeng H, Tufte E, Nylander G (2003). Behandling av mastitt i allmennpraksis. Treatment of mastitis in general practice. Tidsskr Nor Laegeforen.

[8] Mastitis in breastfeeding women Patient leaflets. BMJ Best Practice.

[9] Potter B (2005). Women's experiences of managing mastitis. Community Pract.

[10] Abou-Dakn M, Richardt A, Schaefer-Graf U, Wöckel A (2010). Inflammatory Breast Diseases during Lactation: Milk Stasis, Puerperal Mastitis, Abscesses of the Breast, and Malignant Tumors - Current and Evidence-Based Strategies for Diagnosis and Therapy. Breast Care (Basel).

[11] Witt AM, Bolman M, Kredit S, Vanic A (2016). Therapeutic Breast Massage in Lactation for the Management of Engorgement, Plugged Ducts, and Mastitis. J Hum Lact.

[12] Amir LH, Lumley J (2006). Women's experience of lactational mastitis--'I have never felt worse'. Aust Fam Physician.

[13] Kvist LJ, Larsson BW, Hall-Lord ML (2006). A grounded theory study of Swedish women's experiences of inflammatory symptoms of the breast during breast feeding. Midwifery.

[14] Røseth I, Bongaardt R, Lyberg A, Sommerseth E, Dahl B (2018). New mothers' struggles to love their child. An interpretative synthesis of qualitative studies. Int J Qual Stud Health Well-being.

[15] Kvale S, Brinkmann S, Anderssen TM, Rygge J (2009). Det kvalitative forskningsintervju.

[16] Silverman D (2011). Interpreting qualitative data: a guide to the principles of qualitative research.

[17] Malterud K (2012). Systematic text condensation: a strategy for qualitative analysis. Scand J Public Health.

[18] (2018). WMA DECLARATION OF HELSINKI - ETHICAL PRINCIPLES FOR MEDICAL RESEARCH INVOLVING HUMAN SUBJECTS.

[19] Nortvedt P, Nortvedt F (2018). Smerte: fenomen og etikk.

[20] Cooklin AR, Amir LH, Nguyen CD (2018). Physical health, breastfeeding problems and maternal mood in the early postpartum: a prospective cohort study. Arch Womens Ment Health.

[21] Wambach KA (2003). Lactation mastitis: a descriptive study of the experience. J Hum Lact.

[22] Whelan B, Kearney JM (2015). Breast-feeding support in Ireland: a qualitative study of health-care professionals' and women's views. Public Health Nutr.

[23] Currie L, Richens Y (2009). Exploring the perceptions of midwifery staff about safety culture. Br J Midwifery.

[24] Severinsson E, Holm A (2015). Patients’ Role in Their Own Safety—A Systematic Review of Patient Involvement in Safety. Open J Nurs.

[25] McFadden A, Gavine A, Renfrew MJ (2017). Support for healthy breastfeeding mothers with healthy term babies. Cochrane Database Syst Rev.

[26] Britton C, McCormick FM, Renfrew MJ, Wade A, King SE (2007). Support for breastfeeding mothers. Cochrane Database Syst Rev.

[27] (2014). Nytt liv og trygg barseltid for familien: Nasjonal faglig retningslinje for barselomsorgen.

[28] Rørtveit K, Sætre Hansen B, Leiknes I, Joa I, Testad I, Severinsson E (2015). Patients’ experiences of trust in the patient-nurse relationship—a systematic review of qualitative studies. Open J Nurs.

[29] (2018). Fødsel: erfaringer med fødsels- og barselomsorgen.

[30] Rance S, McCourt C, Rayment J (2013). Women's safety alerts in maternity care: is speaking up enough?. BMJ Qual Saf.

